# A Happy New Year

**Published:** 1890-01-04

**Authors:** 


### Words of Consolation.

### A HAPPY NEW YEAR.

*(For Reading to the Sick.)*

How many times during the last few days have these words been said! In love, they have let the happiness of youth overflow its fellow creatures; in carelessness, they have been used as a matter of fashion; in sarcasm, perhaps, by the warped and disappointed. There are, however, few either so old or so joyless that the thought of a new year does not bring to the mind the possibility of something pleasant happening before the end—a fresh start in life, in which we will leave behind all our old troubles and worries; a fresh skein of life to wind, in which we mean never to have either knots or tangles. These milestones of time on which we rest every 365 days give us an opportunity for thinking of the past and of the future. Of the past, what lost opportunities, what bad habit, what sinful thoughts we have left behind us; we cannot count up the sins we have committed. Alas! they lie thick and threefold "as sands upon the great sea shore." They are more in number than the hairs of our head. We may say with David, "who can tell how oft he offendeth; oh, cleanse Thou me from my secret thoughts." A very good use in which to put the last few days of the old year is to search our hearts and know them, to gather up the fragments that remain of time, health, money, talents, that nothing may be lost, to number our days and apply our hearts unto wisdom. And now let us see to what advantages we can turn this New Year which is lying all before us, wherein we expect so much. We have been ill, we hope to get well; we have had misfortunes, the luck may turn; we have done foolish things, or worse, we have made bad acquaintances, who are leading us on the downward path. Here are the opportunities for improvement, here may be the turning points in our lives. With this fair New Year we feel cheerful and strong, and able to throw aside the sin which so easily besets us, strong and willing to make new resolutions, to break with old ties that keep us back. The discontented thoughts, the peevish manner, the hasty, cutting, angry words are all things to be remembered and avoided in our scheme of improvement, for they blight alike the lives of those who indulge in them, and those who are the victims of the ill tempers. We must not forget, however, in the warmth and cheerfulness of our hearts that we cannot leave ourselves behind with the old year. Each carries his own *ego*, his own particular character about with him wherever he goes, and though at first it seems easy to pass by the wine when it sparkles in the cup, to avoid the wicked who are lying in wait to deceive, to check the angry word or wrong desire, yet by slow degrees the yoke becomes too hard to bear, and we chafe at the restraint our own will exercises; the new year is becoming much like the old, the skein is getting into all the old snarls and tangles. And we are so foolish as to think we can set things straight for ourselves. Unless the grace of God steps in to help, we are but as straws tossed by every wind. He alone can help. He alone can strengthen, confirm, establish us. He knows our weakness, and as a father pitieth his own children, so the Lord hath pity on us. We will turn to Him, then, in repentance and prayer, yielding up our own wills into His hands. So with thankful hearts for all the mercies we have received in the past and the gracious promises for the future, we may ensure to ourselves a happy because it will be a godly New Year.

